# Susceptibility to *Xylella fastidiosa* and functional xylem anatomy in *Olea europaea*: revisiting a tale of plant–pathogen interaction

**DOI:** 10.1093/aobpla/plab027

**Published:** 2021-05-21

**Authors:** Giai Petit, Gianluca Bleve, Antonia Gallo, Giovanni Mita, Giuseppe Montanaro, Vitale Nuzzo, Dario Zambonini, Andrea Pitacco

**Affiliations:** 1 Department of Land, Environment, Agriculture and Forestry (LEAF/TESAF), University of Padua, Viale dell’Università 16, 35020 Legnaro (PD), Italy; 2 Institute of Sciences of Food Production, National research Council (ISPA-CNR), via Provinciale Lecce-Monteroni 73100 Lecce, Italy; 3 Department of European and Mediterranean Culture (DiCEM), University of Basilicata, Via Lanera, 20, 75100 Matera, Italy; 4 Department of Agronomy, Food, Natural resources, Animals and Environment (DAFNAE), University of Padua, Viale dell’Università 16, 35020 Legnaro (PD), Italy

**Keywords:** Cavitation, embolism, hydraulic failure, metabolism, *Olea europaea*, olive quick decline syndrome, OQDS, *Xylella fastidiosa*, xylem

## Abstract

*Xylella fastidiosa* is a xylem-limited bacterium causing the Olive Quick Decline Syndrome, which is currently devastating the agricultural landscape of Southern Italy. The bacterium is injected into the xylem vessels of leaf petioles after the penetration of the insect vector’s stylet. From here, it is supposed to colonize the xylem vasculature moving against water flow inside conductive vessels. Widespread vessel clogging following the bacterial infection and causing the failure of water transport seemed not to fully supported by the recent empirical xylem anatomical observations in infected olive trees. We tested the hypothesis that the higher susceptibility to the *X. fastidiosa*’s infection in Cellina di Nardò compared with Leccino is associated to the higher vulnerability to air embolism of its larger vessels. Such hypothesis is motivated by the recognized ability of *X. fastidiosa* in degrading pit membranes and also because air embolism would possibly provide microenvironmental conditions more favourable to its more efficient aerobic metabolism. We revised the relevant literature on bacterium growth and xylem physiology, and carried out empirical field, mid-summer measurements of xylem anatomy and native embolism in olive cultivars with high (Cellina di Nardò) and low susceptibility (Leccino) to the infection by *X. fastidiosa*. Both cultivars had similar shoot mass traits and vessel length (~80 cm), but the highly susceptible one had larger vessels and a lower number of vessels supplying a given leaf mass. Native air embolism reduced mean xylem hydraulic conductance by ~58 % (Cellina di Nardò) and ~38 % (Leccino). The higher air-embolism vulnerability of the larger vessels in Cellina di Nardò possibly facilitates the *X. fastidiosa*’s infection compared to Leccino. Some important characteristics of the vector–pathogen–plant interactions still require deep investigations acknowledging both the pathogen metabolic pathways and the biophysical principles of xylem hydraulics.

## Teaser

The olive cultivars Cellina di Nardò and Leccino have different susceptibility to the Olive Quick Decline Syndrome caused by the xylem-limited bacterium *Xylella fastidiosa*. The analysed infected branches of Cellina di Nardò had fewer and larger vessels, and showed higher occurrence of air embolism (higher percent loss of xylem conductance, PLC) in the field compared to those of Leccino. Results would suggest that air embolism in xylem vessels likely plays a key role in predisposing the host plant to a more virulent pathogen attack.

## Introduction

*Xylella fastidiosa* is a gram-negative, non-flagellated bacterium highly specialized to colonize xylem vessels of several host plant species. Recently, *X. fastidiosa* subsp. *pauca* has been accidentally introduced in Salento (Apulia, Southern Italy) (Saponari *et al.* 2013), where it is causing the olive quick decline syndrome (OQDS), which has already devastated the olive groves of Salento region and is now continuously expanding, threatening all the Mediterranean region (Godefroid *et al.* 2019). The degree of the OQDS symptoms due to the infection of *X. fastidiosa* might greatly differ among olive cultivars to the extent that some of them (including the cv Leccino) appear to tolerate the infection (Martelli 2016). However, the mechanisms behind such a tolerance remain still not fully explored.

### Insect vector, pathogen and host plant interactions

There is a general agreement that insect vectors hosting cells of *X. fastidiosa* in their foregut inoculate these bacterial cells into the xylem vasculature while feeding on the leaf petioles of the host plant, from where it spreads into the xylem of branches and stem (Almeida 2016). Since xylem vessels conduct water from roots to leaves, *X. fastidiosa* is supposedly able to spread along the xylem vasculature by efficiently moving against sap flow (Meng *et al.* 2005; Chen and De La Fuente 2020).

A common consequence of the plant–pathogen interaction is the clogging xylem vessels by aggregates of tyloses and gelspossibly limiting the pathogen spread (Pearce 1996).

### Insect vectors and environmental conditions at the sites of inoculation

Xylem-feeding insects, such as sharpshooters leafhoppers (Hemiptera, Cicadellidae) and spittlebugs (Hemiptera, Cercopidae), can spread the pathogen *X. fastidiosa* by inoculating the bacterium into the xylem vessels when feeding from infected to not-infected plants. In the Mediterranean, the spittlebug *Philaenus spumarius* has been recognized as the main vector of *X. fastidiosa* (Cornara *et al.* 2017). This insect can host bacterial cells in its foregut, and may release them into the xylem transport system of the host plants when inserting its stylet into the leaf petiole up to the vessel elements to feed on xylem sap (Backus *et al.* 2015; Cornara *et al.* 2018). *Xylella fastidiosa* will then infect host plants by entering the xylem vasculature while mixed with the insect’s salivary egestion (Backus *et al.* 2015). Despite the stylet penetration into the cell wall implies a significant mechanical damage, it has been argued that salivary egestion can prevent vessel embolization by efficient wound sealing, allowing these specialized insects to feed on xylem sap even under high tension (Malone *et al.* 1999). From one hand, the assumption that such a physical damage to the vessel cell wall does not cause air entry into a vessel with sap pressure being easily at 1 MPa below atmospheric pressure would appear rather unlikely to many plant physiologists. On the other, it is certainly unrealistic that the vessel does not embolize upon stylet retraction, as its lumen would be directly connected with the external atmosphere (see below for details on air-embolism formation). Consequently, it seems arguable that vessels just infected by *X. fastidiosa* cannot remain hydraulically functional.

### Bacterial colonization in xylem and spread through pit membranes

According to a functional *in silico* study, *X. fastidiosa* has a very simple and unusual respiratory complex and can use it at high aeration levels, whereas anaerobic respiration is limited to the use of sulfur metabolism (Bhattacharyya *et al.* 2002). Similar results were obtained with analyses of genetic, phenotypic and computational data, suggesting an incomplete anaerobic respiration metabolic network, coupled with a functional and more efficient, although limited, aerobic respiratory system (Gerlin *et al.* 2020).

During plant colonization, bacterial cells are in a planktonic plant-competent status and are able to move along the xylem. Concerning the pathogen propagation in planta, open vessels of primary xylem crossing leaf-branch junctions have been hypothesized to facilitate the pathogen spreading (Chatelet *et al.* 2006). However, the colonization of the xylem vasculature requires the movement of bacteria between adjacent vessels across pit membranes (Newman *et al.* 2003; Cardinale *et al.* 2018). Since the pore diameter in pit membranes are in the range of 30–100 nm (Zhang *et al.* 2020), while the body size of *X. fastidiosa*’s cells are in the range of 250–2400 nm (Wells *et al.* 1987), the activation of cell wall-degrading enzymes is of fundamental importance to enlarge the pores allowing the bacteria to move to adjacent vessels and diffusely spread along the xylem vasculature (Pérez-Donoso *et al.* 2010). Indeed, the presence of high level of diffusible signal factors (DSF) induce the bacteria to synthesize and release cell wall-degrading polygalacturonase and endo-1,4-b-glucanase able to enlarge pores among adjacent xylemic vessels and to allow bacterial cell to pass from one vessel to another (Roper *et al.* 2007; Ionescu *et al.* 2014). Accordingly, the production of surface adhesins and DSF were suggested to relate to the pathogen virulence (Chatterjee *et al.* 2008; Ionescu *et al.* 2014)

### Pit porosity and vulnerability to air embolism

The enlargement of pit pores likely has serious negative consequences on water transport linked to the formation and spread of air emboli.

In plants, the water ascending along the xylem conduits is at sub-atmospheric pressure, and therefore in a metastable phase (i.e. at negative pressure, also called tension: Ψ_*XYL*_). This means that it remains liquid while it should spontaneously change into its vapour state. According to the Laplace law, the maximum diameter of a dissolved air bubble (*d*_*MAX*_) is:


$$\begin{array}{l} d_{MAX}=-4\gamma /\Psi_{XYL} \end{array}$$ (1)

where *γ* is the surface tension at the liquid/air interface at 20 °C: 7.28 × 10^–8^ MPa m). It follows that pit membranes represents a safe barrier against air embolism, as they filter out air bubbles with diameter larger than pores (30–100 nm, Zhang *et al.* 2020). Therefore, the xylem vulnerability to air embolism is directly proportional to the pore size of pit membranes (Jansen *et al.* 2009).

### Xylem anatomy and vulnerability to *X. fastidiosa*

Although vessel occlusions are widely diffused in the xylem of infected plants, their effect on the plant–pathogen interaction is still debated.

According to one hypothesis, more vulnerable plants are less efficient in clogging vessels with tyloses, thus having a limited capacity to compartmentalize the host pathogen (Deyett *et al.* 2019). Instead, an opposite hypothesis would be that the loss of conductance due to vessel occlusions by bacterial aggregates and tyloses produces detrimental consequences on leaf water supply (Hopkins 1989; Sun *et al.* 2013). Consistent with the above hypotheses, a higher vulnerability to *X. fastidiosa*’s infection would be expected either in plants with larger vessel due to the larger volume of vasculature to be filled with tyloses for pathogen compartmentalization, or in plants with narrower vessels because the more efficient clogging of their lumina would more severely limit water transport.

Notably, empirical evidence suggested that the typical desiccation symptoms of *X. fastidiosa*’s infection commonly occur prior to tylose formation (Pérez-Donoso *et al.* 2016), thus implicitly questioning the causal relationship between vessel occlusions and disease symptoms. Furthermore, the appearance of worst disease symptoms is not necessarily associated with widely diffused bacterial aggregates or tyloses occluding vessels. In fact, *X. fastidiosa* cells can sparsely diffuse on vessel cell walls without producing lumen occlusions (Gambetta *et al.* 2007; De Benedictis *et al.* 2017; Novelli *et al.* 2019).

Consistent with the ability of *X. fastidiosa* to degrade pit membranes, it has been reported that the loss of xylem hydraulic conductance due to drought-induced air embolism is more severe in infected vs. non-infected plants, and typically precedes the appearance of leaf scorch symptoms (McElrone *et al.* 2008). According to established literature on plant hydraulics, the higher vulnerability to air embolism is commonly associated to larger xylem conduits (Hacke *et al.* 2006). Therefore, it could also be hypothesized that the higher vulnerability to *X. fastidiosa*’s infection in some plants is associated with larger and less air-embolism resistant vessels (Sabella *et al.* 2019).

The aim of this work is to clearly characterize the difference in functional xylem anatomy and allocation patterns between two olive cultivars (Cellina di Nardò and Leccino), characterized by very different symptomatology to *X. fastidiosa*’s infection. We test the hypothesis that the more symptomatic Cellina di Nardò, which grows faster and is more productive than Leccino, has larger and fewer vessels sustaining leaf transpiration, thus possibly being more susceptible to drought-induced air embolism and to the pathogen infection.

## Material and Methods

The study area was located at Alessano (Lecce, Apulia, Italy) (39°54′55″N, 18°19′16″E), where a 1 ha rainfed plantation of *Olea europaea* grove consisted of two sectors with the presence of the cultivar Cellina di Nardò (planting layout of 7.5 × 7.5 m) and Leccino (planting layout of 4.5 × 4.5 m). Tree age (~20 years) and size (tree height *H* ~ 6–7 m) was similar across individuals. All olive trees showed clear symptoms of infection by *X. fastidiosa*, with desiccated leaf area accounting for >70 % of the total canopy in Cellina di Nardò and <30 % in Leccino (by visual estimate). Due to the widespread diffusion of *X. fastidiosa*’s infection in the area, it was not possible to find a comparative *X. fastidiosa*-free site or with completely asymptomatic plants to be used as control. Sampling took place between the 9th and 14th of July 2019. Details of climate conditions during the season and at the time of field sampling are shown in **Supporting Information—**Fig. S1.

### Measurements of leaf/branch biomass and xylem anatomy

**Figure 1.**
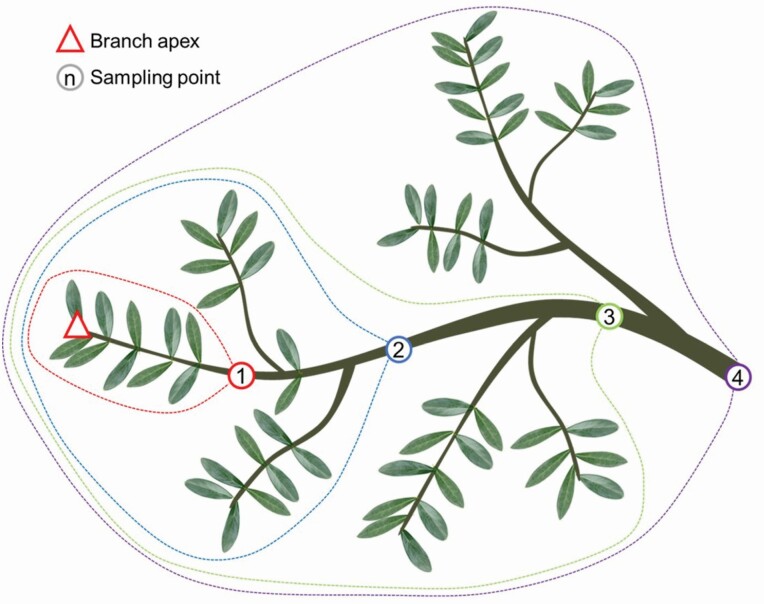
Branch sampling: sampling points (numbered circles) were identified along the main branch axis, and the distance from the branch apex (*DFA*) measured. The dotted lines identify the total leaf biomass (*LM*_*CUM*_) and the total xylem mass, *BM*_*CUM*_) distal to each sampling point (i.e. cumulated from the branch apex).

We cut a sun exposed branch sample of ~2 m without visual signs of infection from the apical part of the crown of five tree for both cultivars. In each branch, we identified 4–5 sampling points distributed at increasing distance from the apex downward along the main branch axis (Fig. 1), and for each point we measured the distance from the branch apex (*DFA*). Starting from the branch apex, we measured the total leaf biomass (*LM*_*CUM*_) and the total branch biomass (*BM*_*CUM*_, including the bark) that cumulated down to each sampling point (i.e. between the branch apex and the given sampling point: see dotted lines in Fig. 1). Leaf and xylem biomass were measured as dry weight after 48 h at 60 °C in the oven.

Furthermore, at each sampling position we extracted a segment of ~1.5 cm for the anatomical analyses. Segment ends were trimmed with a sharp blade to clearly visualize the cross-sectional surface free of sawdust, and then were observed under a stereomicroscope to check for the presence of vessel occlusions. Afterwards, they were put in a pressure cooker under vapour pressure for 1 h to soften the xylem tissues, and then cut with a rotary microtome (Leica RM2245; Leica Biosystems, Nussloch, Germany) at 15–20 μm. Sections were stained with safranine and AstraBlue (1 and 0.5 % in distilled water, respectively), and slides permanently fixed with Eukitt (BiOptica, Milan, Italy). Section images were acquired at 100× magnification, using a D-sight slide scanner (Menarini Group, Florence, Italy), and analysed with ROXAS (von Arx and Carrer 2014). The analysis was performed on a wedge of known angle (*α*, between 10 and 40 degrees) centred at the pith. Among many different parameters, the software automatically measured the lumen area of each individual vessel (*VA*) and calculated the total number of vessels (*VN*), the mean vessel lumen diameter (*VLD*, simplifying vessels as cylinders with length of 1 mm) and the total hydraulic conductivity (*Kh*) (based on the Hagen-Poiseuille law: Tyree and Ewers 1991). Data of *VN* and *Kh* were finally multiplied by 360/*α* to rescale the ROXAS outputs to the total cross-sectional area of the sample.

### Hydraulic measurements

The maximum vessel length (*VL*_*MAX*_) was estimated on one asymptomatic branch from 5–6 trees per cultivar. Sun exposed branches of 1.5–2 m were excised and immediately transported to the lab into a black plastic bag containing a moist paper. In the lab, the branch base was re-cut under water to remove the basal 20 cm, and the cut surface trimmed with a sharp blade. The branch basal end, with bark removed for ~2 cm, was then attached to a source of degassed Levissima® mineral water, naturally containing several elements in ionic form as in xylem sap (Nardini *et al.* 2007), and flushed at a pressure *P* = 0.2 MPa for 15 min to remove all gas emboli. After flushing, we injected air at low pressure (*P* = 0.02 MPa). The main branch axis was then cut under water starting from the apex downwards approximately every 2 cm. *VL*_*MAX*_ was then estimated as the length of the remaining branch segment once a stream of air bubbles appeared at the apical cut surface.

The assessment of native air embolism was carried out on a sun exposed and asymptomatic branch from 5 to 6 trees per cultivar sampled before 8.00 a.m., i.e. before the development of low leaf water potentials. Immediately after excision, sampled branches were closed into a black plastic bag containing a moist paper to saturate vapour content and to limit leaf gas exchanges, and then transported to the lab. Hydraulic measurements were carried out after a sufficient time (>30 min) to re-equilibrate the water potential gradients inside the branches. Relaxed branches were then progressively cut under water starting from the base for a length of >80 cm (i.e. >*VL*_*MAX*_), thus removing long vessels possibly embolized upon branch excision in the field (Torres-Ruiz *et al.* 2015). The apical part of the branch axis was also cut under water in order to obtain a branch segment of length >*VL*_*MAX*_. Both the apical and basal cut surfaces were trimmed under water with a sharp razor blade to remove ~1 mm. The branch base, with bark removed for a length of ~2 cm, was then attached to a source of degassed Levissima® mineral water with a pressure head of ~0.02 MPa. The water flow rate (*F*, g s^−1^) was then estimated based on the weight of water collected at the apical end of the branch pressurized for a period of 3 min into an Eppendorf vial containing a dry sponge of known weight. We repeated three measurements per sample. The initial branch conductance (*Ki*) was then calculated as *F*/*P*. Branch samples were then flushed at *P* = 0.2 MPa for 15 min to remove all gas emboli, and then remeasured three times at *P* = 0.02 MPa (maximum branch conductance, *Kmax*). The percentage loss of hydraulic conductance (*PLC*) was then calculated as:


$$\begin{array}{l} PLC=\left(1-\frac{Ki}{Kmax}\right)\times 100 \end{array}$$ (2)

### Estimate of bacterial contamination

We estimated the presence of *X. fastidiosa* cells in a subset of leaves detached from the branches sampled for the measurements of biomass allocation and xylem anatomy, and in the water extracted from the xylem during the hydraulic measurements.

Petioles and leaf basal parts were sampled from 20 leaves of each branch and pulverized in liquid nitrogen. The resulting material was processed for CTAB-based DNA extraction according to the procedure reported in EPPO Bulletin focussed on diagnostics of *X. fastidiosa* (2019). Specifically, 0.5–1 g of fresh material were homogenized with 10 volumes of CTAB buffer (CTAB 2 %, Tris–HCl pH 8 100 mM, EDTA 20 mM, NaCl 1.4 M, PVP-40 1 %). An extract aliquot (1 mL) was incubated at 65 °C for 30 min and after centrifugation to remove plant tissue debris (16 000 g for 10 min), the supernatant was treated with chloroform: isoamyl alcohol (24:1) and total nucleic acids were isolated by precipitation with 2-propanol. After washing with 70 % ethanol, the pelleted DNA was resuspended in 100 µL of TE buffer.

During the hydraulic measurements, the water flowing at *P* = 0.02 MPa was collected from branches before (500 µL) and after (2 mL) flushing at *P* = 0.2 MPa. Samples were centrifuged at 8000g × 5 min to collect bacterial cells and the pellet was resuspended in 45 µL of NaOH 50 mM. After boiling for 10 min, 5 µL of Tris–HCl 1 M were added. In the case of water collected after flush at *P* = 0.2 MPa, a further phenol: chloroform extraction step was performed, followed by precipitation and washing in ethanol. The pelleted DNA was resuspended in 100 µL of TE buffer.

DNA concentration was evaluated by absorbance at 260 nm and concentration of samples was adjusted to 100 ng µL^−1^. Absolute quantification of *X. fastidiosa* in plants was performed by qPCR (Harper *et al.* 2010).

The assay was performed on the Applied Biosystem Step One system by adding 2 µL of DNA sample in a reaction volume of 20 µL containing ITaq Probe Master Mix 2X (Biorad). Each sample was run in triplicate. A standard calibration curve was used based on DNA extracted from 10-fold dilutions of health olive extract spiked with a bacterial suspension with an initial OD 600 of 0.5, corresponding to about 10^8^ CFU mL^−1^.

### Statistical analyses

Structural and anatomical traits of stem and branches are known to vary longitudinally along the main axis of stem and branches. In order to account for the strict dependence of these traits on the distance from the distal apex (*DFA*) (Anfodillo *et al.* 2013; Petit *et al.* 2016), we tested for the differences between cultivars in several allometric scaling relationships:


$$\begin{array}{l} Y=a\times X^{b} \end{array}$$ (3)

Data were first Log_10_-transformed to accomplish for the assumption of normality and homoscedasticity (Zar 1999), so that equation (3) becomes:


$$\begin{array}{l} Log_{10}Y=Log_{10}a+b\times Log_{10}X \end{array}$$ (4)

We then tested differences between cultivars using linear mixed-effects models fitted with restricted maximum likelihood (REML) using the lme4 package (Bates *et al.* 2015) of the software R (R Development Core Team 2014). We used *DFA*, *Cultivar* and their interaction as fixed effects, and tree ID as random factor in all initial models. The best model was chosen based on Akaike Information Criterion (AIC) using the maximum likelihood method (Zuur *et al.* 2009).

## Results

### Branch biomass allocation and xylem anatomy

The sampled asymptomatic branches of both Cellina di Nardò and Leccino revealed no presence of vessel cloggings upon observations at the stereoscope.

**Figure 2.**
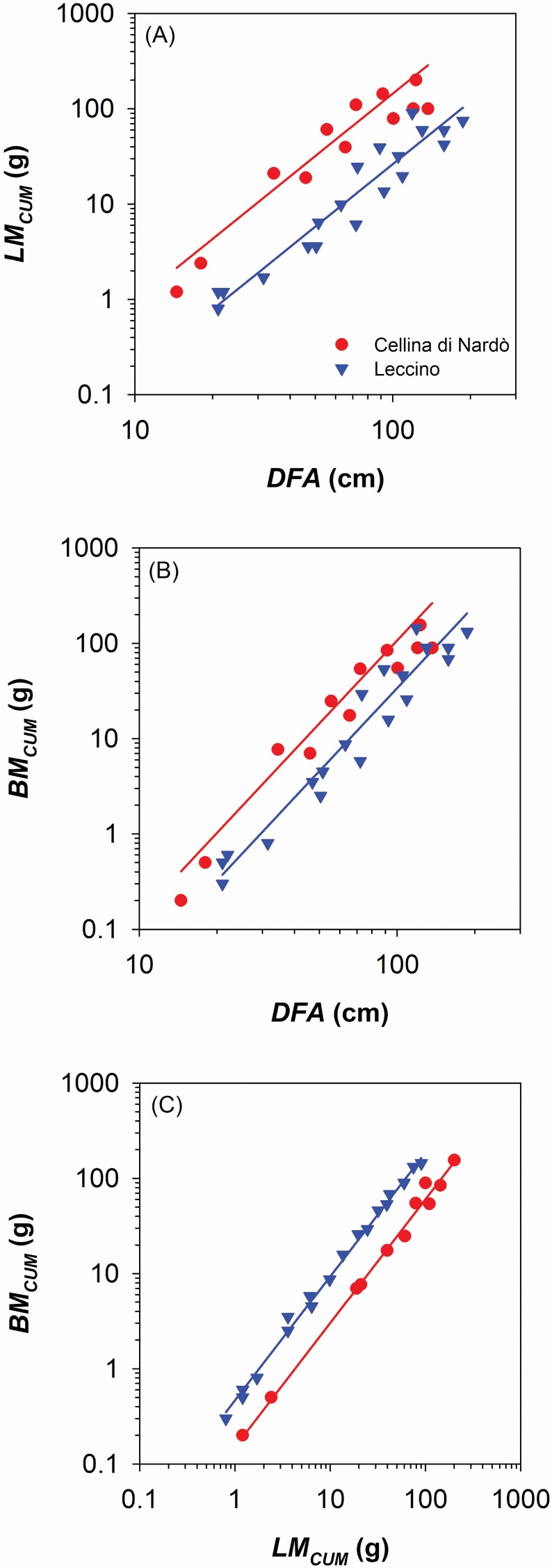
(A) Relationship between total leaf area (i.e. cumulated starting from the branch apex, *LM*_*CUM*_) and distance from the branch apex (*DFA*). (B) Relationship between total branch biomass (i.e. cumulated starting from the branch apex, *BM*_*CUM*_) and *DFA*. (C) Relationship between *BM*_*CUM*_ and *LM*_*CUM*_. Axes are displayed with logarithmic scale and parameters of fitting lines for Cellina di Nardò (red circles) and Leccino (blue triangles) are according to Table 1.

**Figure 3.**
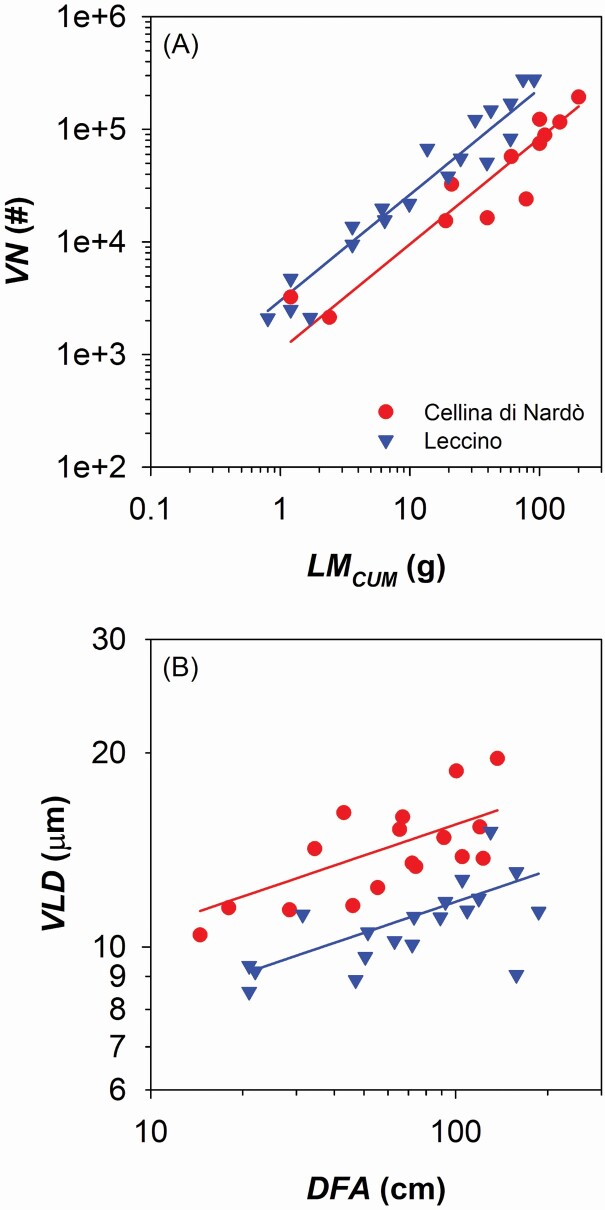
(A) Relationship between number of vessels (*VN*) and total supplied leaf mass (*LM*_*CUM*_). (B) Variation in vessel lumen diameter (*VLD*) and distance from the branch apex (*DFA*). Axes are displayed with logarithmic scale and parameters of fitting lines for Cellina di Nardò (red circles) and Leccino (blue triangles) are according to Table 1.

**Figure 4.**
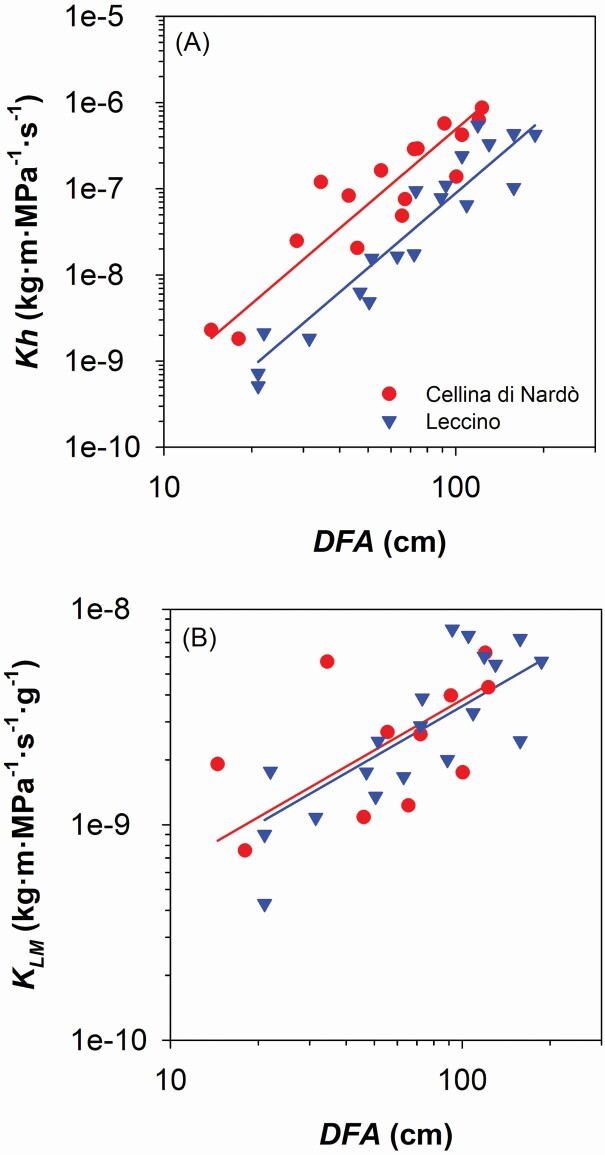
(A) Relationship between xylem hydraulic conductivity (*Kh*) and distance from the branch apex (*DFA*). (B) Relationship between leaf-mass specific hydraulic conductivity (*K*_*LM*_) and *DFA*. Axes are displayed with logarithmic scale and parameters of fitting lines for Cellina di Nardò (red circles) and Leccino (blue triangles) are according to Table 1.

We found that Cellina di Nardò allocated more biomass along the branch axis than Leccino. Moving from the branch apex downwards along the main branch axis, Cellina di Nardò cumulated more leaf mass (*LM*_*CUM*_) than Leccino (i.e. higher *y*-intercept in the relationship of *LM*_*CUM*_ vs. distance from the apex, *DFA*: Fig. 2A; Table 1), but had not significantly higher xylem mass (*BM*_*CUM*_) (i.e. not significantly different *y*-intercept in the relationship of *BM*_*CUM*_ vs. *DFA*: Fig. 2B; Table 1). Consequently, Cellina di Nardò distributed along the branch less xylem in proportion to leaf biomass compared with Leccino (Fig. 2C; Table 1), and consistently produced a lower number of vessels (*VN*) connected to the distal leaf mass (i.e. lower *y*-intercept in the relationship of *VN* vs. *LM*_*CUM*_: Fig. 3A; Table 1). However, Cellina di Nardò hydraulically compensated the lower *VN* associated to a given leaf mass by producing vessels with significantly larger lumen diameter (*VLD*) (i.e. higher *y*-intercept in the relationship of *VLD* vs. *DFA*: Fig. 3B; Table 1; example images are shown in **Supporting Information—**Fig. S2). In fact, although the hydraulic conductivity (*Kh*) was higher in Cellina di Nardò compared with Leccino (i.e. higher *y*-intercept in the relationship *Kh* vs. *DFA*: Fig. 4A; Table 1), the scaling of leaf-mass specific conductivity (*K*_*LM*_ = *K*_*H*_*/LM*_*CUM*_) vs. *DFA* was not significantly different between the two cultivars (Fig. 4B; Table 1), i.e. the absolute branch conductivity necessary to supply a unit of distal leaf mass does not significantly differ between the two cultivars.

**Table 1.** Statistics of the selected linear mixed-effects models predicting the difference between cultivars (Cellina di Nardò and Leccino, with the former taken as reference) on different relationships between traits describing allocational (A), anatomical (B) and functional (C) patterns. Plant ID was used as random factor.

	Model	Covariates and fixed effects	Estimate ± SE	DF	*t*-value	*P*	*R* ^2^m	*R* ^2^c
(A) ALLOCATION	*A1) LOG*_*10*_ *LM*_*CUM*_ ~ *LOG*_*10*_*DFA + Cultivar* (+*ID*_*PLANT*_)	Intercept (y0)	−2.30 ± 0.20	24	−11.75	<0.0001	0.91	0.97
		Slope (β)	2.19 ± 0.08	24	26.22	<0.0001		
		Cultivar (LE)	−2.95 ± 0.17	3	−3.89	0.0301		
	*A2) LOG*_*10*_ *BM*_*CUM*_ ~ *LOG*_*10*_*DFA + Cultivar* (+*ID*_*PLANT*_)	Intercept (y0)	−3.89 ± 0.21	24	−18.65	<0.0001	0.93	0.98
		Slope (β)	2.90 ± 0.08	24	36.01	<0.0001		
		Cultivar (LE)	−4.26 ± 0.20	3	−1.87	0.1589		
	*A3) LOG*_*10*_ *BM*_*CUM*_ ~ *LOG*_*10*_*LM*_*CUM*_ + *Cultivar* (+*ID*_*PLANT*_)	Intercept (y0)	−0.82 ± 0.04	24	−20.03	<0.0001	0.99	0.99
		Slope (β)	1.31 ± 0.02	24	76.50	<0.0001		
		Cultivar (LE)	−0.34 ± 0.04	3	11.70	0.013		
(B) ANATOMY	*B1) LOG*_*10*_ *NV ~ LOG*_*10*_ *LM*_*CUM*_ + *Cultivar* (+*ID*_*PLANT*_)	Intercept (y0)	3.04 ± 0.11	24	26.85	<0.0001	0.90	0.91
		Slope (β)	0.94 ± 0.05	24	17.37	<0.0001		
		Cultivar (LE)	3.48 ± 0.10	3	4.28	0.0234		
	*B2) LOG*_*10*_ *VLD ~ LOG*_*10*_ *DFA + Cultivar* (+*ID*_*PLANT*_)	Intercept (y0)	0.87 ± 0.05	29	15.83	<0.0001	0.67	0.68
		Slope (β)	0.16 ± 0.03	29	5.24	<0.0001		
		Cultivar (LE)	0.75 ± 0.02	4	−6.68	0.0026		
(C) FUNCTION	*C1) LOG*_*10*_ *Kh ~ LOG*_*10*_ *DFA + Cultivar* (+*ID*_*PLANT*_)	Intercept (y0)	−12.17 ± 0.30	28	40.38	<0.0001	0.86	0.93
		Slope (β)	2.92 ± 0.19	28	19.93	<0.0001		
		Cultivar (LE)	−12.87 ± 0.20	4	−3.14	0.0347		
	*C2) LOG*_*10*_ *K*_*LM*_ ~ *LOG*_*10*_*DFA + Cultivar* (+*ID*_*PLANT*_)	Intercept (y0)	−10.00 ± 0.26	24	−38.34	<0.0001	0.48	0.58
		Slope (β)	0.79 ± 0.14	24	5.73	<0.0001		
		Cultivar (LE)	−10.02 ± 0.13	3	−0.14	0.9		

### Xylem air embolism

The maximum vessel length was similar along the distal 2 m of branches in both cultivars (*VL*_*MAX*_ ~ 80 cm; Table 2).

**Table 2.** Mean values (standard deviation between parentheses) of measured maximum vessel length (*VL*_*MAX*_), percentage loss of hydraulic conductance (*PLC*), count of *X. fastidiosa* cells in the leaves (*XF*_*LEAF*_) and in the water flowing through the xylem vessels during the hydraulic measurements (at low pressure:*XF*_*FUNCT. VESSELS*_; during perfusion at high pressure: *XF*_*EMB. VESSELS*_) (n.d.: under limit of detection of 10^2^ CFU mL^−1^). *Estimated excluding sample C6 = 2.8 × 10^2^ CFU mL^−1^.

*Cultivar*	*VL*_*MAX*_ (cm)	*PLC* (%)	*XF*_*LEAF*_ (CFU mL^−1^ × 10^3^)	*XF*_*FUNCT. VESSELS*_ (CFU mL^−1^)	*XF*_*EMB. VESSELS*_ (CFU mL^−1^)
Cellina di Nardò	80.8 (5.5)	58.0 (21.7)	35.9 (31.6)	n.d.	n.d.*
Leccino	75.7 (12.7)	37.9 (5.6)	9.61 (8.2)	n.d.	n.d.

The analyses on the diffusion of gas emboli filling vessels in *in vivo* plants (known as native air embolism) through the assessment of the percentage loss of xylem conductance (*PLC*) in living branches in the field revealed a significantly higher *PLC* (*P* = 0.04) in Cellina di Nardò (*PLC* = 58.0 %) than Leccino (*PLC* = 37.9 %) (Table 2).

### Bacterial contamination of leaves and branch xylem

The analyses revealed the presence of *X. fastidiosa* subsp. *pauca* cells in leaf samples from asymptomatic branches of both cultivars, but more abundant in Cellina di Nardò than Leccino (*P* = 0.008) (Table 2). Furthermore, the presence of the bacterium in functional vessels of asymptomatic branches was estimated by collecting the outflow during the measurements of *Ki* at low pressure (*P* = 0.02 Pa), and from the previously embolized vessels by collecting the water during the flushing at high pressure (*P* = 0.2 Pa). In both cases, it was not possible to determine the presence of bacteria likely because they were below the minimum detectable value (10^2^ CFU mL^−1^) (Table 2). However, for one out of four Cellina di Nardò samples, we found *X. fastidiosa* cells (2.8 10^2^ CFU mL^−1^) only after removing the gas emboli by flushing the sample at high pressure.

## Discussion

Our results revealed clear differences in functional xylem anatomy and allocation patterns between the olive cultivars Cellina di Nardò and Leccino. In natural field conditions, these cultivars showed different susceptibility to the infection by *X. fastidiosa* (Martelli 2016). Since inoculation is dependent on the feeding activity of the insect vector *P. spumarius* (Cornara *et al.* 2017), the different symptomatology between olive cultivars likely depends on intrinsic differences in structure/function relationships and/or response/defensive mechanisms to the pathogen attack.

Trees used in this experiment showed leaf scorch symptoms in various sectors of the crown denoting the bacteria infection, but also the sampled asymptomatic branches were infected as proved by the significant level of bacterial contamination at the leaf level (Table 2). We can, therefore, assume that asymptomatic infected branches were sampled before the appearance of desiccations symptoms and possibly before the formation of vessel occlusions.

Cellina di Nardò (highly susceptible) produced shoots with more leaf and branch biomass compared with Leccino. However, the theoretical efficiency in leaf water supply did not differ between cultivars, because larger vessels would compensate for the reduced number of vessels per leaf mass in Cellina di Nardò compared to Leccino. Consequently, embolization of single vessels would be more limiting to water transport in Cellina di Nardò.

Vessels increased in diameter from the branch apex downwards (Fig. 3B), according to a pattern that is extremely stable across years (Anfodillo *et al.* 2013; Prendin *et al.* 2018). Because of this pattern, most of the total hydraulic resistance would be concentrated within the very last/apical portion of branches (Yang and Tyree 1993; Lechthaler *et al.* 2020), and the contribution of the narrower vessels of inner rings would be much less important than that of the outermost one.

Substantially, we did not observe vessel occlusions in our 1–2-year-old branches. Seemingly, a recent study reported a very low percentage of occluded vessels in the current (<2 %) and previous year (2–5 %) xylem rings of branches of both Cellina di Nardò and Leccino, and only in older rings the percentage increased at 15–20 % and 5–10 %, respectively (Sabella *et al.* 2020).

Notably, such a magnitude of vessel occlusions unlikely would determine a percentage loss of xylem conductance seriously limiting the leaf water supply. Typically, *X. fastidiosa* produces aggregates into xylem vessels of petioles (Sun *et al.* 2013; Sabella *et al.* 2019), but the bacterial colonization further down along the branch vasculature is less intense (Baccari and Lindow 2010). Furthermore, occlusions most often are caused by tyloses and gels produced by the plant rather than the direct proliferation of bacterial cells (De Benedictis *et al.* 2017; Sabella *et al.* 2019), thus questioning the relationship between the degree of leaf scorch symptoms and the abundance of *X. fastidiosa* cells in the xylem vasculature of the host plant (Gambetta *et al.* 2007; De Benedictis *et al.* 2017).

Our limited empirical hydraulic data on native embolism would suggest that Cellina di Nardò is more vulnerable to air embolism than Leccino (Table 2). Notably, the highest *PLC* of ~84 % was recorded in some branches of Cellina di Nardò. Such degrees of xylem hydraulic dysfunction have been argued to seriously compromise the plant survival, with foliar colour changes typically lagging behind hydraulic failure (Urli *et al.* 2013; Hammond *et al.* 2019). However, these data did not prove that the vulnerability to air embolism increased following the progression of the infection.

The higher xylem vulnerability to air embolism in infected Cellina di Nardò compared to Leccino could be related either to simple intrinsic differences in xylem anatomical structures, like the larger vessel diameters (Fig. 3B), or possibly to an amplification effect by *X. fastidiosa* degrading pit membranes.

In this context, future studies comparing whole vulnerability curves of branches from infected vs. non-infected trees in the more symptomatic vs. the less symptomatic olive cultivars possibly will provide a clarifying information on the potential role of air embolism in the aetiology of the infection by *X. fastidiosa*.

In the simplest case that infected olive trees were not more vulnerable to air embolism than non-infected ones, than the higher air-embolism vulnerability in Cellina di Nardò could likely predispose this cultivar to a more virulent infection by *X. fastidiosa* compared to Leccino, especially under drought conditions (McElrone *et al.* 2003), as typically occurs in the study area **[see****Supporting Information—**Fig. S1**]**.

A worse case scenario would be if infected olive trees were more vulnerable to air embolism than non-infected ones. This situation could be further corroborated by anatomical analyses on pit membrane integrity, as high pit membrane porosity is associated to higher xylem vulnerability to air embolism (Wheeler *et al.* 2005). Indeed, previous observations revealed pit membrane degradation following the digestion of the cellulosic and hemicellulosic components of the plant cell walls (Roper *et al.* 2007). Damages on pit membranes were reported to facilitate the bacterium spread to neighbouring vessels (McElrone *et al.* 2008; Pérez-Donoso *et al.* 2010).

Although not tested in our study, and to date never been accounted for in previous studies, we push forward the hypothesis that xylem embolism could play a key role in the aetiology of the infection by *X. fastidiosa*, possibly providing more favourable conditions for its preferred and more efficient aerobic respiration.

Indeed, *X. fastidiosa* can perform both limited anaerobic and aerobic respiration (Bhattacharyya *et al.* 2002). However, in its metabolic network there is a functional and preferred aerobic respiration, since no complete anaerobic respiration was found to be functional (Gerlin *et al.* 2020). Furthermore, functional vessels represent a difficult environment for life under water: water is under very negative pressure with low oxygen concentration, as big-sized air bubbles are filtered out by the small pores of pit membranes (Zhang *et al.* 2020).

We tried a novel hydraulic method to test for the presence of *X. fastidiosa* in air-embolized vs. functional vessels. Notably, a relatively small but significant number of *X. fastidiosa* cells was found only in air-embolized vessels form one branch of Cellina di Nardò. However, bacteria cells could possibly strongly adhere the infected xylem walls, thus limiting the removal potential of perfusing wood samples both at high and low pressure. A possible methodological improvement to better quantify the presence of *X. fastidiosa* in functional vs. air-embolized vessels could be the use of biofilm disrupting compounds increasing the *X. fastidiosa* release from vessel cell walls.

We push forward also the hypothesis that air embolism likely occur at the moment of *X. fastidiosa* inoculation while vector insects feed on leaf petioles. Based on the known laws of physics, a great mechanical damage to the vessel cell wall would be produced as the insect vector inserts the stylet into the petiole tissues. This would cause the water tension to be immediately released, reaching atmospheric pressure by the suction of air into the lumen, thus possibly providing aerobic conditions during the first phases of infection.

In conclusion, we provided evidence for clear differences in the functional xylem anatomy between the two olive cultivars Cellina di Nardò and Leccino. The more *X. fastidiosa* symptomatic Cellina di Nardò appeared to be more vulnerable to drought-induced embolism formation because of its larger and fewer vessels. Since the magnitude of vessel clogging in infected olive trees appeared unlikely to provide hydraulic limitations to water transport (Sabella *et al.* 2020), we encourage the scientific community to further investigate on the potential relationship between xylem air embolism and the metabolic activity of the xylem-limited pathogen *X. fastidiosa*.

## Supporting Information

The following additional information is available in the online version of this article—

**Figure S1.** Daily value of maximum temperature (Tmax), precipitations (Prec) and reference evapotranspiration (ET0, calculated according to the Hargreaves equation: Allen et al. 1998) during the year 2019.

**Figure S2.** Sample images of the xylem anatomy showing the axial variation.

plab027_suppl_Supplementary_Materials

plab027_suppl_Supplementary_Data

## Data Availability

Data are publicly available as **Supporting Information** and at the public data repository of the University of Padua (http://researchdata.cab.unipd.it/id/eprint/489).

## Notes

**Form & Function. Chief Editor:** Kate McCulloh
