# Supplementary material for: Susceptibility to Xylella fastidiosa and functional xylem anatomy in Olea europaea: revisiting a tale of plant–pathogen interaction
Source: AoB Plants. 2021 May 21;13(4):plab027. doi: 10.1093/aobpla/plab027 (PMC8300559; doi:10.1093/aobpla/plab027)
Supplement: plab027_suppl_Supplementary_Materials [file plab027_suppl_supplementary_materials.pdf]

SUPPLEMENTARY INFORMATION

Susceptibility to *Xylella fastidiosa* and functional xylem anatomy in *Olea europaea*: revisiting a tale of plant-pathogen interaction

Giai Petit[1]*, Gianluca Bleve[2], Antonia Gallo[2], Giovanni Mita[2], Giuseppe Montanaro[3], Vitale Nuzzo[3], Dario Zambonini[1] and Andrea Pitacco[4]

[1]Dept. of Land, Environment, Agriculture and Forestry (LEAF / TESAF), University of Padua, Viale dell'Università 16, 35020 Legnaro (PD), Italy

[2] Institute of Sciences of Food Production, National research Council (ISPA-CNR), via Provinciale Lecce-Monteroni

73100 Lecce, Italy

[3]Dept. of European and Mediterranean Culture (DiCEM), University of Basilicata, Via Lanera, 20, 75100 Matera, Italy

[4]Dept. of Agronomy, Food, Natural resources, Animals and Environment (DAFNAE), University of Padua, Viale dell'Università 16, 35020 Legnaro (PD), Italy

* Corresponding Author: giai.petit@unipd.it

Figure S1

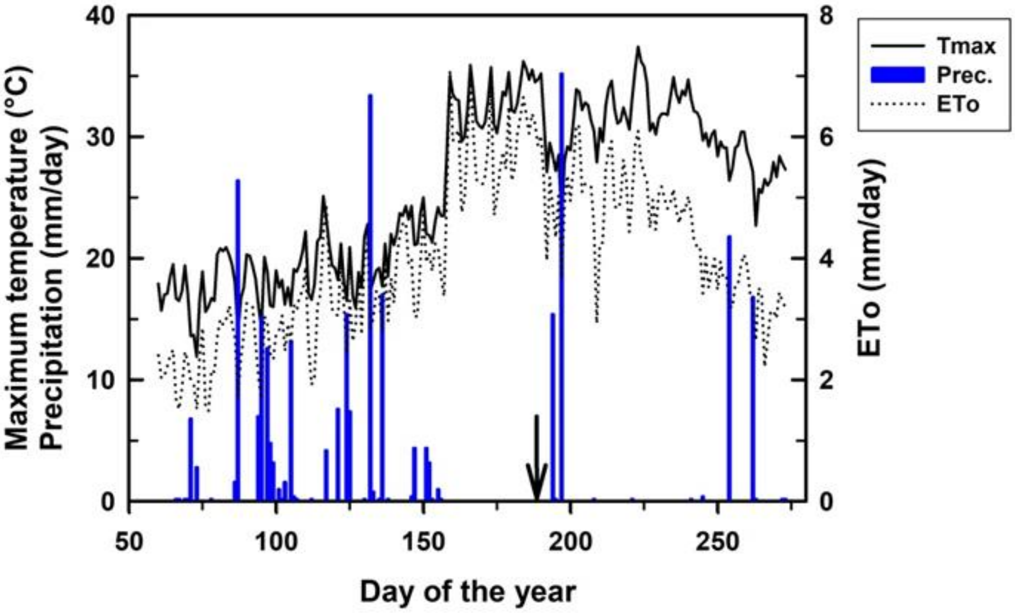

Daily value of maximum temperature (Tmax), precipitations (Prec) and reference evapotranspiration (*ET_0*, calculated according to the Hargreaves equation: Allen *et al.*, 1998) during the year 2019. Data were recorded from an automatic standard weather station (39.88417 N, 18.31722 E), belonging to the National Civil Protection Department located near the experimental field site. The arrow indicates the timing of field sampling and hydraulic analyses.

Figure S2

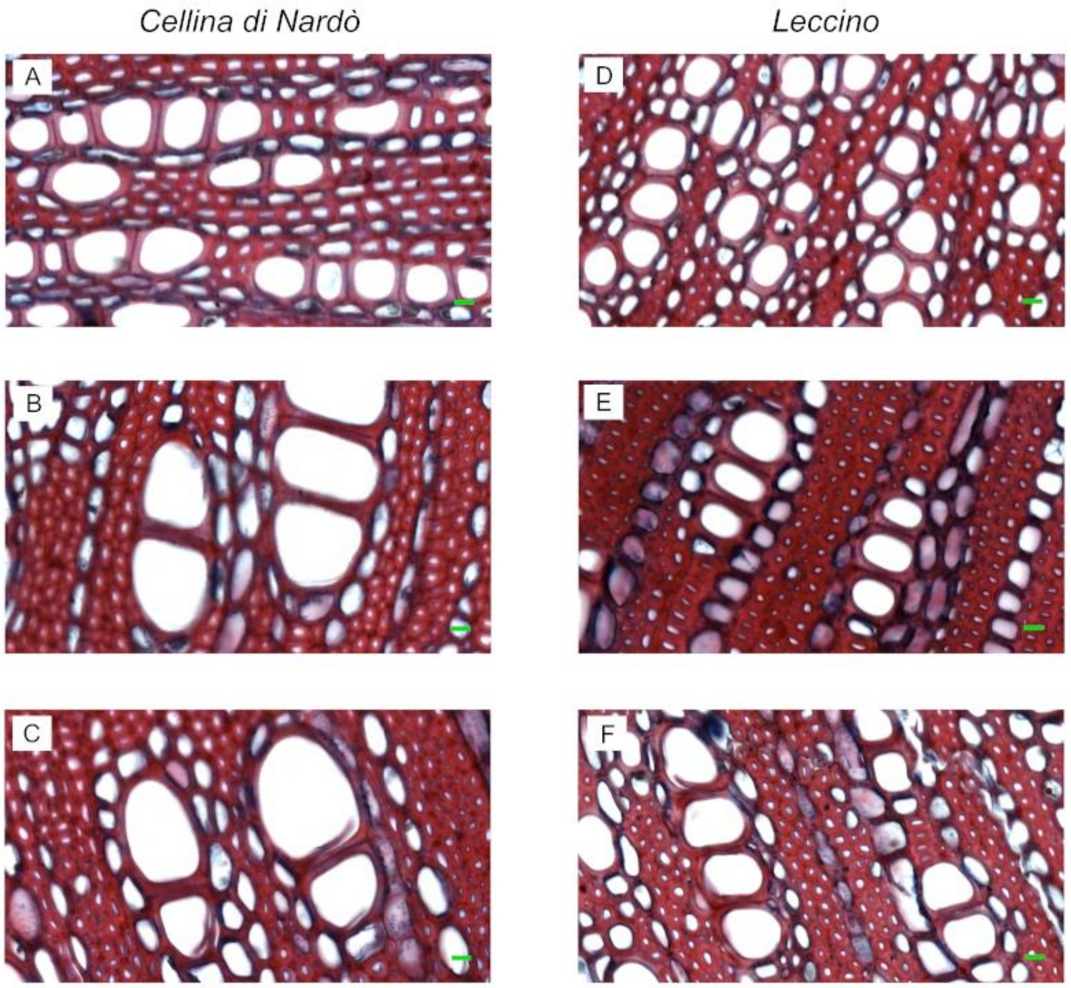

Sample images of the xylem anatomy showing the axial variation taken at increasing distance from the branch apex (*DFA*) in Cellina di Nardò (A, B, C) and in Leccino (D, E, F). *DFA* is 20 cm in (A, D), 75 cm in (B, E) and 105 cm in (C, F). Scale bar is 10 µm.

References

Allen RG, Pereira SL, Raes D, Smith M. **1998**. Crop evapotranspiration (guidelines for computing crop water requirements). *FAO Irrigation and Drainage Paper* **56**.
